# Supplementary material for: Targeting APC/C Ubiquitin E3-Ligase Activation with Pyrimidinethylcarbamate Apcin Analogues for the Treatment of Breast Cancer
Source: Biomolecules. 2024 Nov 12;14(11):1439. doi: 10.3390/biom14111439 (PMC11591962; doi:10.3390/biom14111439)
Supplement: Supplementary file 1 [file biomolecules-14-01439-s001.zip › biomolecules-3253073-Supplementary Information.docx]

Supplementary material.

1. General Chemical Synthesis Strategy

1.1 Development of the West-East route. Preparation of APN (e.g., compound **1**), Cbz-APN (e.g., compound **2**), and Me-APN (e.g., compound **3**)**.**

The preparation of APN analogues **1**, **2** and **3** was accomplished through the synthetic sequence shown in **Figure S1**, following the method outlined in the literature [13].


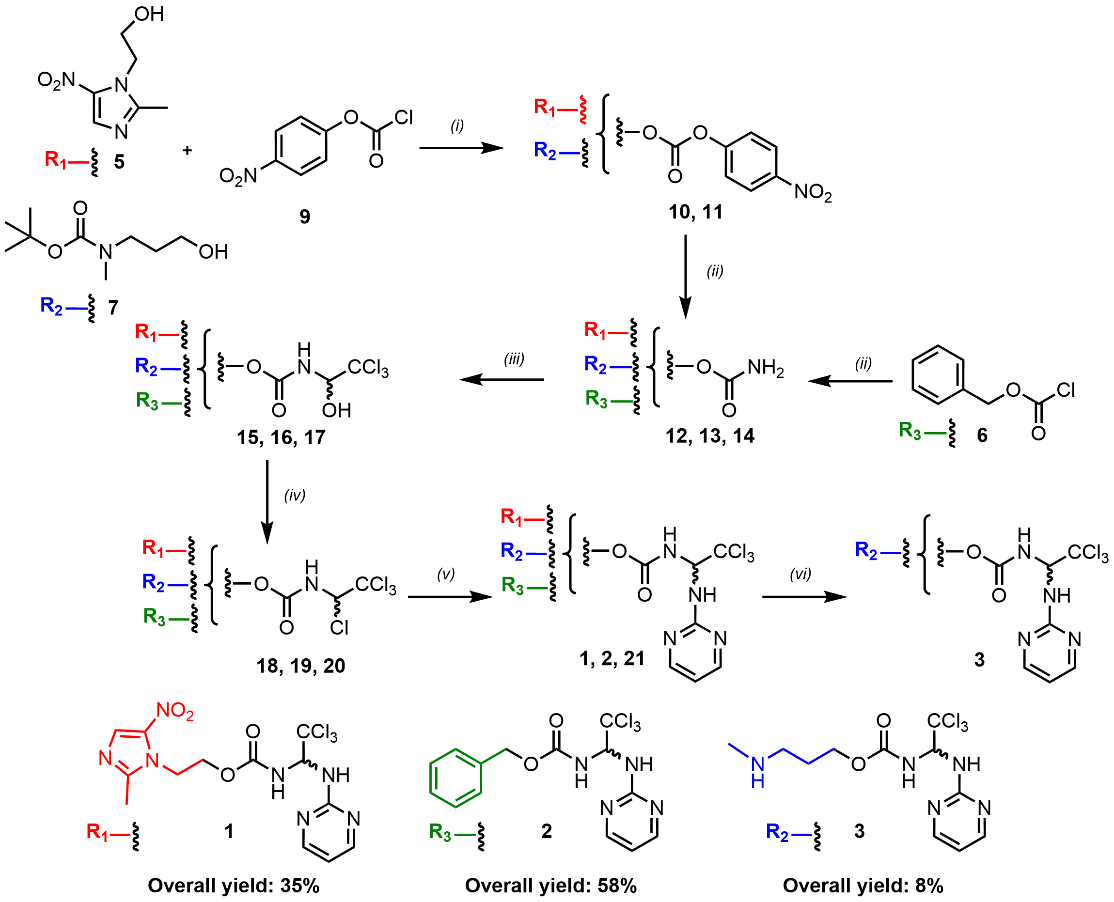


**Figure S1**. Synthetic route for preparation of APN (**1**), Cbz-APN (**2**) y Me-APN (**3**). Reaction Conditions: (*i)* Et_3_N, CH_2_Cl_2_, 0 °C → r.t.; (*ii)* NH_4_OH, MeOH, 0 °C → 10 °C; (*iii)* Chloral hydrate, r.t. → 100 °C; (*iv)* SOCl_2_, Py, r.t. → reflux; (*v)* 2-aminopyrimidine, Et_3_N, CH_3_CN, r.t. → reflux, (*vi)* CF_3_COOH, CH_2_Cl_2_, r.t.

The synthesis of APN (**1**) and its analogue Me-APN (**3)** following the *West-East Route* began with the functionalisation of the hydroxyl group of metronidazole (**5**) and 3-[(*tert*-butoxycarbonyl) methylamino]propan-1-ol (**7**) [38] to generate nitrophenylcarbonates **10** and **11**, respectively, which subsequently led to the formation of the carbamate group present in compounds **1** and **3**. This was achieved by reacting them with 4-nitrophenyl chloroformate **9**, and through their subsequent treatment with an aqueous solution of ammonia in methanol, carbamates **12** and **13** were formed [17]. For the preparation of carbamate **14**, a different synthetic approach was followed; in this, the reaction of benzyl chloroformate **6** and an aqueous solution of ammonia in methanol led to the formation of the desired compound [39,40]. The introduction of the trichloromethyl group was carried out using chloral hydrate by heating this reagent and compounds **12**, **13**, and **14** in the absence of solvent, which resulted in the formation of alcohols **15**, **16**, and **17**.

The thermodynamic reaction conditions used would allow the formation of a racemic mixture, obtaining both enantiomers. This may be because, under the reaction conditions employed, heating can lead to the in situ formation of trichloroacetaldehyde from chloral hydrate, the actual reactive species [41]. The subsequent nucleophilic attack of the carbamate nitrogen group of compounds **12**, **13**, and **14** on either the *Re* or *Si* face of trichloroacetaldehyde will result in the generation of both enantiomers of compounds **15**, **16**, and **17** since there is no effective facial stereoselectivity (**Figure S2**).

**Figure S2.** Facial diastereoselectivity using chloral hydrate as the alkylating agent.

The synthesis continues with the introduction of the characteristic pyrimidine moiety of compounds with an APN-like structure. For this purpose, it was necessary to activate the hydroxyl group of compounds **15**, **16**, and **17** using thionyl chloride in the presence of a catalytic amount of pyridine [42]. This reaction led to the formation of alkyl chlorides **18**, **19**, and **20**, which were used in the next synthetic step without purification. The reaction of these compounds with 2-aminopyrimidine in the presence of Et_3_N resulted in the formation of the desired products APN **1** and Cbz-APN **2**, as well as carbamate **21** [43]. To prepare the remaining synthetic target through this synthetic route, Me-APN **3**, an additional deprotection step using the standard conditions described in the literature for such reactions will be necessary. To obtain the free amine, an ion-exchange chromatography using DOWEX^®^ 50WX4 acid resin was required.

Through this synthetic sequence, using the *West-East Route*, the preparation of APN **1** has been achieved, improving the existing yield reported in the literature. Additionally, the preparation of two APN structure analogues, Cbz-APN **2**, and Me-APN **3**, has also been achieved. All three compounds have been synthesized with good overall yields: APN **1**, 35%; Cbz-APN **2**, 58%; and Me-APN **3**, 8%. It is worth noting that the yield of the latter could be improved.

1.2. Development of the East-West Route. Preparation of Bzn-APN **4**

The preparation of the final synthetic target of this work, Bzn-APN **4**, was carried out using the East-West Route. As mentioned earlier, this route, being a divergent pathway, allows for the preparation of a wide variety of Apcin analogues from a common synthetic intermediate, chloride **23**. This intermediate serves as a structural diversity point in our synthetic strategy, which is strategically valuable as it enables the introduction of any organic moiety allowed by innovations in organic chemistry (**Figure S3**).

**Figure S3.** Structural diversity of East-West Route.

According to the proposed synthetic scheme and the established work plan (**Figure S4**), 2-aminopyrimidine (**8**) was used as the starting material, which is a common and commercially available product [44]. The heating of amine **8** and chloral hydrate under reflux in THF led to the formation of alcohol **22**. The use of a prochiral reagent along with the reaction conditions employed, as in the West-East Route, will result in a racemic mixture.

**Figure S4.** Synthetic route for the preparation of Bzn-APN **4**. Reaction conditions: (*i)* Chloral hydrate, THF r.t.  reflux; (*ii)* SOCl_2_, dixane, r.t.  reflux; *(iii)* 2-(aminomethyl)benzimidazole dihydrochloride, Et_3_N, CH_3_CN, r.t.

The subsequent treatment of compound **22** with thionyl chloride under reflux in dioxane resulted in alkyl chloride **23**, which was used in the following step without purification. For the synthesis of Bzn-APN **4**, 2-(aminomethyl)benzimidazole dihydrochloride was employed. An additional amount of Et_3_N was necessary to release the amine from the reagent used so that the subsequent substitution reaction could take place. In this way, the synthesis of benzimidazole **4**, the final synthetic target of this work, has been achieved with a good overall yield of 46% in three synthetic steps.

## APN synthesis

### Synthesis of *O*-(4-nitrophenyl)-*O*ʹ-[2-(1*H*-2-methyl-5-nitroimidazol-1-yl)ethyl] carbonate (**10**)

Over a suspension of 2-(1*H*-2-methyl-5-nitroimidazol-1-yl)ethan-1-ol (**5**) (1 g, 5.84 mmol) in dry CH_2_Cl_2_ (11.5 mL), Et_3_N (0.99 mL, 7.01 mmol) was added at room temperature. Then, at 0 °C, 4-nitrobenzyl chloroformate (**9**) (1.21 g, 5.84 mmol) was added. The reaction mixture was stirred at room temperature for 21 hours. After this time, CH_2_Cl_2_ (10 mL) was added to the solution. The resulting organic phase was washed with saturated NaCl (3 × 10 mL). The combined organic extracts were dried with anhydrous Na_2_SO_4_, filtered, and the solvent was removed under reduced pressure. The residue was purified by column chromatography (Hex/EtOAc, 1:4), yielding *O*-(4-nitrophenyl)-*O*ʹ-[2-(1*H*-2-methyl-5-nitroimidazol-1-yl)ethyl]carbonate (**10**) as a yellowish solid (1.96 g, yield 100%).

***R_f_*** = 0.30 (TLC, Hex/EtOAc, 1:4). **^1^H-NMR** (*500 MHz*, *(CD_3_)_2_CO*): δ 8.32 (2H, dm, *J* = 9.3 Hz), 7.94 (1H, s), 7.48 (2H, dm, *J* = 9.3 Hz), 4.84 (2H, dd, *J* = 5.6, 4.4 Hz), 4.73 (2H, dd, *J* = 5.6, 4.4 Hz), 2.55 (3H, s) ppm. **^13^C-NMR** (*125 MHz*, *(CD_3_)_2_CO*): δ 156.4, 153.0, 152.4, 146.6, 139.8, 133.6, 126.1, 123.2, 68.1, 45.6, 14.4 ppm. **IR** (*KBr*): ν 3423, 1778, 1616, 1591, 1525, 1423, 1350, 1237, 1210, 1180, 1108, 1054, 820 cm^−1^.

### Synthesis of [2-(1*H*-2-methyl-5-nitroimidazol-1-yl)ethyl]carbamate (**12**)

Over a suspension of *O*-(4-nitrophenyl)-*O*ʹ-[2-(1*H*-2-methyl-5-nitroimidazol-1-yl)ethyl] carbonate (**10**) (2.55 g, 7.58 mmol) in MeOH (39 mL), a 4M solution of NH_3_ (3.40 mL, 53.08 mmol) was slowly added dropwise at 0 °C. The reaction mixture was stirred at 10 °C for 2 hours. After this time, the solvent was removed under reduced pressure. The residue was purified by column chromatography (EtOAc/MeOH, 50:1), yielding [2-(1*H*-2-methyl-5-nitroimidazol-1-yl)ethyl]carbamate (**12**) (1.21 g, yield 81%) as a white solid.

***R_f_*** = 0.10 (TLC, EtOAc). **^1^H-NMR** (*500 MHz*, *(CD_3_)_2_CO/DMSO-d6 [10:1]*): δ 7.93 (1H, s), 6.33 (2H, b.s.), 4.59 (2H, dd, *J* = 5.7, 4.6 Hz), 4.36 (2H, dd, *J* = 5.7, 4.6 Hz), 2.50 (3H, s) ppm. **^13^C-NMR** (*125 MHz*, *(CD_3_)_2_CO*): δ 156.9, 152.4, 139.4, 133.4, 62.6, 46.4, 14.3 ppm. **IR** (*KBr*): ν 3379, 3280, 3181, 2731, 1726, 1621, 1536, 1476, 1429, 1366, 1328, 1191, 1147, 1130, 1108, 1086, 1004, 982, 858, 741 cm^−1^.

### Synthesis of *rac*-[2-(1*H*-2-methyl-5-nitroimidazol-1-yl)ethyl]-*N*-(2,2,2-trichloro-1-hydroxyethyl)carbamate (**15**)

A mixture of [2-(1*H*-2-methyl-5-nitroimidazol-1-yl)ethyl]carbamate (**12**) (1.21 g, 5.66 mmol) and 2,2,2-trichloroethane-1,1-diol (1.00 g, 5.95 mmol) was heated at 100 °C for 19 hours. After that time, the reaction mixture was dissolved in MeOH (30 mL). Subsequently, the solvent was removed under reduced pressure. The residue was purified by column chromatography (EtOAc/MeOH, 50:1), affording *rac*-[2-(1*H*-2-methyl-5-nitroimidazol-1-yl)ethyl]*-N*-(2,2,2-trichloro-1-hydroxyethyl)carbamate (**15**) (1.18 g, yield 58%) as a white solid.

***R_f_*** = 0.35 (TLC, EtOAc). **^1^H-NMR** (*500 MHz*, *(CD_3_)_2_CO*): δ 7.90 (1H, s), 7.49 (1H, d, *J* = 9.5 Hz), 6.79 (1H, d, *J* = 5.7 Hz), 5.64 (1H, dd, *J* = 9.5, 5.7 Hz), 4.65 (2H, t, *J* = 5.0 Hz), 4.62-4.56 (1H, m), 4.50-4.40 (1H, m), 2.48 (3H, s) ppm. **^13^C-NMR** (*125 MHz*, *(CD_3_)_2_CO*): δ 156.0, 152.5, 139.6, 133.5, 102.7, 84.7, 64.0, 46.5, 14.4 ppm. **IR** (*KBr*): ν 3418, 3060, 2846, 2709, 1726, 1536, 1498, 1465, 1429, 1369, 1294, 1264, 1215, 1191, 1149, 1106, 1045, 828, 812 cm^−1^.

### Synthesis of *rac*-[2-(1*H*-2-methyl-5-nitroimidazol-1-yl)ethyl]*-N*-[2,2,2-tricloro-1-(pirimidin-2-ylamino)ethyl]carbamate (**1**)

Over a suspension of *rac*-[2-(1*H*-2-methyl-5-nitroimidazol-1-yl)ethyl]-*N*-(2,2,2-trichloro-1-hydroxyethyl)carbamate (**15**) (1.18 g, 3.27 mmol) in dry CH_2_Cl_2_ (13 mL), Py (70 μL, 0.82 mmol) was added at room temperature. The reaction mixture was stirred at 10 °C for 20 minutes. After that time, SOCl_2_ (0.29 mL, 3.93 mmol) was added at room temperature, and the solution was refluxed in CH_2_Cl_2_ for 2 hours. Subsequently, the solvent was removed under reduced pressure, affording the intermediate *rac*-[2-(1*H*-2-methyl-5-nitroimidazol-1-yl)ethyl]-*N*-(1,2,2,2-tetrachloroethyl)carbamate (**18**), which was used in the next step without further purification.

Over a mixture of *rac*-[2-(1*H*-2-methyl-5-nitroimidazol-1-yl)ethyl]*-N*-(1,2,2,2-tetrachloroethyl)carbamate (**18**) (1.56 g, 4.11 mmol) and 2-aminopyrimidine (403 mg, 4.11 mmol) in CH_3_CN (20 mL), Et_3_N (0.87 mL, 6.16 mmol) was added at room temperature. The reaction was refluxed in CH_3_CN for 18 hours. After that time, the solvent was removed under reduced pressure. The residue was purified by column chromatography (Hex/EtOAc, 1:5), yielding *rac*-[2-(1*H*-2-methyl-5-nitroimidazol-1-yl)ethyl]-*N*-[2,2,2-trichloro-1-(pyrimidin-2-ylamino)ethyl] carbamate (**1**) (1.07 g, yield 75%) as a white solid.

***R_f_*** = 0.33 (TLC, EtOAc/MeOH, 10:1). **^1^H-NMR** (*500 MHz*, *DMSO-d6*): δ 8.42 (2H, d, *J* = 4.8 Hz), 8.01 (1H, d, *J* = 9.3 Hz), 7.99 (1H, s), 7.12 (1H, d, *J* = 9.3 Hz), 6.83 (1H, t, *J* = 4.8 Hz), 6.61 (1H, t, *J* = 9.3 Hz), 4.53 (2H, t, *J* = 5.0 Hz), 4.50-4.44 (1H, m), 4.38-4.32 (1H, m), 2.38 (3H, s) ppm. **^13^C-NMR** (*125 MHz*, *DMSO-d6*): δ 160.4, 158.3, 154.8, 151.6, 138.4, 133.1, 113.0, 102.3, 69.6, 63.0, 45.4, 13.9 ppm. **LRMS** (*API-ES^+^*): *m/z* 438 (M+H)^+^, 460 (M+Na)^+^. **IR** (*Solid*): ν 3414, 3186, 3122, 2977, 1729, 1688, 1584, 1536, 1487, 1443, 1426, 1364, 1349, 1326, 1258, 1190, 1153, 1110, 1055, 1012, 996, 971, 901, 826, 797, 764, 744, 683, 617, 580, 527, 492, 419 cm^−1^.

## Cbz-APN synthesis

### Synthesis of (benzyl)carbamate (**14**)

Over a solution of 4 M NH_3_ (18.60 mL, 293.10 mmol) in MeOH (60 mL), benzyl chloroformate (**6**) (4.40 mL, 29.31 mmol) was added at 0 °C. The reaction mixture was stirred at 0 °C for 1 hour. After that time, the solvent was removed under reduced pressure. The residue was purified by column chromatography (Hex/EtOAc, 1:1), yielding (benzyl)carbamate (**14**) (3.783 g, 85% yield) as a white solid.

***R_f_*** = 0.55 (TLC, Hex/EtOAc, 1:1). **^1^H-RMN** (*300 MHz*, *CDCl_3_*): δ 7.38-7.32 (5H, m), 5.11 (2H, s), 4.67 (2H, b.s.) ppm.

### Synthesis of benzyl-(2,2,2-trichloro-1-hydroxyethyl)carbamate (**17**)

A mixture of (benzyl)carbamate (**14**) (3.78 g, 2.03 mmol) and 2,2,2-trichloroethane-1,1-diol (7.60 g, 45.05 mmol) was heated to 100 °C for 23 hours. After that time, the reaction mixture was dissolved in MeOH (30 mL). Subsequently, the solvent was removed under reduced pressure. The residue was purified by column chromatography (Hex/EtOAc, 5:1), yielding *rac*-benzyl-(2,2,2-trichloro-1-hydroxyethyl)carbamate (**17**) (6.23 g, 83% yield) as a white solid.

***R_f_*** = 0.50 (TLC, Hex/EtOAc, 2:1). **^1^H-NMR** (*400 MHz*, *CDCl_3_*): δ 7.36-7.27 (5H, m), 5.85 (1H, d, *J* = 9.2 Hz), 5.71 (1H, d, *J* = 9.2 Hz), 5.45 (1H, s_ancho_), 5.13 (2H, s) ppm. **^13^C-NMR** (*100 MHz*, *CDCl_3_*): δ 155.2, 135.4, 128.5, 128.3, 128.1, 100.9, 83.3, 67.6 ppm. **LRMS** (*EI*): *m/z* 298.9 (M^·+^,1.4), 180.1 (1.4), 108.2 (100).

### Synthesis of *rac*-benzyl-*N*-[2,2,2-trichloro-1-(pyrimidin-2-ylamino)ethyl] carbamate (**2**)

Over a suspension of *rac*-benzyl*-N*-(2,2,2-trichloro-1-hydroxyethyl)carbamate (**17**) (1.17 g, 3.91 mmol) in dry CH_2_Cl_2_ (40 mL), Py (80 μL, 0.98 mmol) was added at room temperature. The reaction mixture was stirred for 20 minutes at 10 °C. After that time, SOCl_2_ (0.35 mL, 4.69 mmol) was added at room temperature. The reaction mixture was refluxed in CH_2_Cl_2_ for 4 hours. Subsequently, the solvent was removed under reduced pressure, yielding the intermediate *rac*-benzyl*-N*-(1,2,2,2-tetrachloroethyl)carbamate (**20**), which was used in the next step without purification.

Over a mixture of *rac*-benzyl-*N*-(1,2,2,2-tetrachloroethyl)carbamate (**20**) (1.24 g, 3.91 mmol) and 2-aminopyrimidine (422 mg, 4.30 mmol) in CH_3_CN (40 mL), Et_3_N (0.83 mL, 5.87 mmol) was added at room temperature. The reaction was refluxed in CH_3_CN for 19 hours. After this time, the solvent was removed under reduced pressure. The residue was purified by column chromatography (Hex/EtOAc, 2:1), yielding *rac*-benzyl-*N*-[2,2,2-trichloro-1-(pyrimidin-2-ylamino)ethyl] carbamate (**2**) (1.21 g, 82% yield) as a white solid.

***R_f_*** = 0.20 (TLC, Hex/EtOAc, 2:1). **^1^H-RMN** (*400 MHz*, *CD_3_OD*): δ 8.36 (2H, d, *J* = 4.7 Hz), 7.39-7.24 (5H, m), 6.84 (1H, s), 6.77 (1H, t, *J* = 4.7 Hz), 5.13 (2H, s) ppm. **^13^C-RMN** (*100 MHz*, *CD_3_OD*): δ 162.1, 159.4, 157.6, 137.8, 129.5, 129.1, 128.9, 113.9, 103.2, 71.4, 68.2 ppm. **LRMS** (*API-ES^+^*): *m/z* 375 (M+H)^+^, 397 (M+Na)^+^. **IR** (*Solid*): ν 3427, 3351, 3314, 3041, 2984, 2924, 2854, 2488, 1723, 1699, 1573, 1527, 1489, 1422, 1410, 1364, 1327, 1259, 1240, 1219, 1180, 1142, 1095, 1054, 1041, 1029, 1014, 971, 947, 903, 814, 798, 785, 736, 673, 640, 617, 593, 529, 464 cm^−1^.

## Me-APN synthesis

### 4.1 Synthesis of 3-[(*tert*-butoxycarbonyl)methylamino]propan-1-ol (**7**)

Over a solution of 3-(methylamino)propan-1-ol (1.15 mL, 10.190 mmol) in CH_2_Cl_2_ (10.0 mL), Et_3_N (1.60 mL, 11.210 mmol) was added at room temperature. Subsequently, at 0 °C, di-tert-butyl dicarbonate (2.80 mL, 12.230 mmol) was added. The reaction mixture was stirred at room temperature for 48 hours. After that time, the reaction mixture was diluted with CH_2_Cl_2_ (50.0 mL). The resulting organic phase was washed with an aqueous solution of 0.1 M HCl (2 × 10.0 mL), H_2_O (1 × 10.0 mL), and an aqueous solution of saturated NaCl (1 × 10.0 mL). Finally, the resulting organic phase was dried over anhydrous MgSO_4_, filtered, and the solvent was removed under reduced pressure, yielding 3-[(*tert*-butoxycarbonyl)methylamino]propan-1-ol (**7**) (1.915 g, yield 100%) as a transparent oil.

***R_f_*** = 0.58 (TLC, EtOAc). **^1^H-RMN** (*300 MHz*, *CDCl_3_*) δ: 3.55 (2H, t, *J* = 5.9 Hz), 3.37 (2H, t, *J* = 5.9 Hz), 2.83 (3H, s), 1.68 (2H, qu, *J* = 5.9 Hz), 1.46 (9H, s) ppm [38].

### 4.2. Synthesis of *O*-{3-[(*tert*-butoxycarbonyl)methylamino]propan-1-yl}-*O*'-(4-nitrophenyl)carbonate (**11**)

Over a solution of 3-[(*tert*-butoxycarbonyl)methylamino]propan-1-ol (**7**) (1.928 g, 10.119 mmol) in CH_2_Cl_2_ (15.0 mL), Et_3_N (1.75 mL, 12.230 mmol) was added at room temperature. Subsequently, at 0 °C, a solution of 4-nitrophenyl chloroformate (2.120 g, 10.190 mmol) in CH_2_Cl_2_ (10.0 mL) was added. The resulting suspension was stirred at 0 °C for 2 hours and then at room temperature for an additional 24 hours. After that time, the reaction mixture was diluted with CH_2_Cl_2_ (25.0 mL). The organic phase was washed with an aqueous solution of saturated NaCl (3 × 10.0 mL). Finally, the resulting organic phase was dried over anhydrous Na_2_SO_4_, filtered and the solvent was removed under reduced pressure. The reaction residue was purified by column chromatography (Hex:EtOAc, 3:1), yielding *O*-{3-[(*tert*-butoxycarbonyl)methylamino] propan-1-yl}-*O*'-(4-nitrophenyl)carbonate (**11**) (1.995 g, 55% yield) as a yellowish oil.

***R_f_*** = 0.23 (TLC, Hex/EtOAc, 3:1). **^1^H-NMR** (*500 MHz*, *CDCl_3_*) δ: 8.25 (2H, dm, *J* = 9.2 Hz), 7.36 (2H, dm, *J* = 9.2 Hz), 4.30 (2H, t, *J* = 6.5 Hz), 3.37 (2H, t, *J* = 6.5 Hz), 2.87 (3H, s), 1.97 (2H, qu, *J* = 6.5 Hz), 1.45 (9H, s) ppm. **^13^C-NMR** (*125 MHz*, *CDCl_3_*) δ: 155.8, 155.6, 152.6, 145.5, 125.4, 121.9, 79.8, 67.1, 45.7 as well as 45.2, 34.4, 28.5, 27.2 and 26.9 ppm [45,46]. **LRMS** (*API-ES^+^*): *m/z* 377 (M+Na)^+^. **IR** (*Solid*): ν 3119, 3084, 2975, 2933, 1765, 1689, 1616, 1594, 1525, 1492, 1393, 1347, 1327, 1255, 1211, 1151, 1110, 1051, 1012, 940, 924, 860, 817, 773, 754, 724, 663, 645, 583, 531, 496, 461, 441 cm^−1^.

### 4.3. Synthesis of {3-[(*tert*-butoxycarbonyl) methylamino]propan-1-yl}carbamate (**13**)

Over a solution of *O*-{3-[(*tert*-butoxycarbonyl)methylamino]propan-1-yl}-*O*'-(4-nitrophenyl)carbonate (**11**) (1.995 g, 5.629 mmol) in MeOH (20.0 mL), an aqueous solution of NH_3_ (3.6 mL, 56.290 mmol) was added dropwise at 0 °C. The yellowish solution formed was stirred at 0 °C for 24 hours. After that time, the solvent was removed under reduced pressure. Toluene (20 mL) was added onto the resulting residue and evaporated under reduced pressure (× 2 times). Finally, the reaction residue was purified by column chromatography (Hex:EtOAc, 2:3), yielding {3-[(*tert*-butoxycarbonyl) methylamino]propan-1-yl}carbamate (**13**) (986 mg, 75% yield) as a white solid.

***R_f_*** = 0.54 (TLC, Hex/EtOAc, 1:2). **^1^H-NMR** (*500 MHz*, *CDCl_3_*) δ: 4.95 (2H, s), 4.03 (2H, t, *J* = 6.5 Hz), 3.26 (2H, t, *J* = 6.5 Hz), 2.81 (3H, s), 1.80 (2H, qu, *J* = 6.5 Hz), 1.41 (9H, s) ppm. **^13^C-NMR** (*125 MHz*, *CDCl_3_*) δ: 157.2, 155.8, 79.5, 62.8, 46.1 as well as 45.6, 34.4, 28.5, 27.5 and 27.2 ppm [45,46]. **LRMS** (*API-ES^+^*): *m/z* 255 (M+Na)^+^.

### 4.4. Synthesis of *rac*-{3-[(*tert*-butoxycarbonyl)methylamino]propan-1-yl}-*N*-[2,2,2-trichloro-1-hydroxyethyl]carbamate (**16**)

A mixture of {3-[(*tert*-butoxycarbonyl)methylamino]propan-1-yl}carbamate (**13**) (936 mg, 4.029 mmol) and 2,2,2-trichloroethane-1,1-diol (816 mg, 4.835 mmol) was stirred at 100 °C for 24 hours. After that time, MeOH (2.0 mL) was added to the reaction mixture followed by celite and the solvent was removed under reduced pressure. The residue was purified by column chromatography (Hex:EtOAc, 3:1), yielding *rac*-{3-[(*tert*-butoxycarbonyl)methylamino]propan-1-yl}-*N*-(2,2,2-trichloro-1-hydroxyethyl)carbamate (**16**) (936 mg, 61% yield) as a colorless oil.

***R_f_*** = 0.19 (TLC, Hex/EtOAc, 1:1). **^1^H-NMR** (*500 MHz*, *CD_3_OD*) δ: 5.59 (1H, b.s.), 4.10 (2H, t, *J* = 6.6 Hz), 3.34 (2H, t, *J* = 6.6 Hz), 2.86 (3H, s), 1.89 (2H, qu, *J* = 6.6 Hz), 1.46 (9H, s) ppm. **^13^C-NMR** (*125 MHz*, *CD_3_OD*) δ: 157.8, 157.6, 103.2, 84.9, 81.1, 64.1 and 63.9, 46.7, 34.8 as well as 34.7, 28.8, 28.1 ppm [45,46]. **LRMS** (*API-ES^+^*): *m/z* 401 (M+Na)^+^. **IR** (*Solid*): ν 3661, 3298, 2974, 2902, 1664, 1486, 1454, 1400, 1395, 1311, 1229, 1152, 1088, 1047, 921, 891, 868, 814, 772, 697, 625, 584, 833, 477, 471, 463, 442, 411 cm^−1^.

### 4.5. Synthesis of *rac*-{3-[(*tert*-butoxycarbonyl)methylamino]propan-1-yl}-*N*-[2,2,2-trichloro-1-(pyrimidin-2-ylamino)ethyl]carbamate (**19**)

Over a solution of *rac*-{3-[(*tert*-butoxycarbonyl)methylamino]propan-1-yl}-*N-*(2,2,2-trichloro-1-hydroxyethyl)carbamate (**16**) (357 mg, 0.940 mmol) in CH_2_Cl_2_ (4.0 mL), pyridine (19.0 µL, 0.235 mmol) was added at 0 °C, and the mixture was stirred at that temperature for 20 minutes. After this period, SOCl_2_ (84 µL, 1.128 mmol) was added at room temperature, and the reaction mixture was heated to reflux in CH_2_Cl_2_ for 2 hours. After this time, the solvent was removed under reduced pressure, yielding *rac*-{3-[(*tert*-butoxycarbonyl)methylamino]propan-1-yl}-*N*-(1,2,2,2-tetrachloroethyl)carbamate (**19**), which was used in the subsequent step without purification.

Next, a solution of *rac*-{3-[(*tert*-butoxycarbonyl)methylamino]propan-1-yl}-*N*-(1,2,2,2-tetrachloroethyl)carbamate (**19**) and 2-aminopyrimidine (92 mg, 0.940 mmol) in CH_3_CN (4.0 mL) was prepared, and Et_3_N (0.20 mL, 1.410 mmol) was added at room temperature. The reaction mixture was refluxed in CH_3_CN for 18.5 hours. After this time, celite was added to the reaction mixture and the solvent was removed under reduced pressure. The residue was purified by column chromatography (Hex:EtOAc, 2:3), yielding *rac*-{3-[(*tert*-butoxycarbonyl) methylamino]propan-1-yl}-*N*-[-2,2,2-trichloro-1-(pyrimidin-2-ylamino)ethyl]carbamate (**21**) (166 mg, 39% yield) as a white solid.

***R_f_*** = 0.16 (TLC, Hex/EtOAc, 1:1). **^1^H-NMR** (*500 MHz*, *CD_3_OD*) δ: 8.39 (2H, d, *J* = 4.9 Hz), 6.84 (1H, b.s.), 6.80 (1H, t, *J* = 4.9 Hz), 4.16-4.06 (2H, m), 3.41-3.26 (2H, m), 2.84 (3H, s), 1.93-1.82 (2H, m), 1.41 (9H, s) ppm. **^13^C-NMR** (*125 MHz*, *CD_3_OD*) δ: 162.1, 159.5, 157.7, 157.6, 113.9, 103.4, 81.0, 71.3, 64.4 and 64.3, 46.7, 34.8 as well as 34.5, 28.7, 28.1 ppm [45,46]. **LRMS** (*API-ES^+^*): *m/z* 356 (M-101)^+^.

### 4.6. Synthesis of *rac*-[3-(methylamino)propan-1-yl]-*N*-[2,2,2-trichloro-1-(pyrimidin-2-ylamino)ethyl]carbamate (**3**)

Over a solution of *rac*-{3-[(*tert*-butoxycarbonyl)methylamino]propan-1-yl}-*N*-[-2,2,2-trichloro-1-(pyrimidin-2-ylamino)ethyl]carbamate (**21**) (166 mg, 0.363 mmol) in CH_2_Cl_2_ (34.0 mL), CF_3_COOH (0.30 mL, 3.634 mmol) was added at room temperature. The solution was stirred at room temperature for 24 hours. After this time, the solvent was removed under reduced pressure. The reaction residue was purified by ion exchange chromatography (H_2_O), yielding *rac*-[3-(methylamino)propan-1-yl]-*N*-[2,2,2-trichloro-1-(pyrimidin-2-ylamino)ethyl]carbamate (**3**) (105 mg, 81% yield) as a yellowish solid.

***R_f_*** = 0.53 (TLC, EtOAc/MeOH/NH_3_, 6:2:1). **^1^H-NMR** (*500 MHz*, *CD_3_OD*) δ: 8.39 (2H, d, *J* = 4.9 Hz), 6.82 (1H, s), 6.80 (1H, t, *J* = 4.9 Hz), 4.25-4.12 (2H, m), 2.66 (2H, t, *J* = 6.0 Hz), 2.38 (3H, s), 1.86 (2H, qu, *J* = 6.0 Hz) ppm. **^13^C-NMR** (*125 MHz*, *CD_3_OD*) δ: 162.1, 159.5, 157.8, 113.9, 103.3, 71.3, 64.6, 49.1, 35.8, 29.5 ppm. **LRMS** (*API-ES^+^*): *m/z* 356 (M+H)^+^. **IR** (*Solid*): ν 3225, 3158, 3064, 2924, 2853, 2801, 2410, 2326, 1725, 1704, 1653, 1616, 1584, 1538, 1504, 1444, 1412, 1378, 1314, 1237, 1184, 1135, 1093, 1046, 995, 897, 866, 815, 796, 774, 729, 662, 641, 628, 581, 519, 474, 467, 419 cm^−1^.

## Bzn-APN synthesis

### Synthesis of *rac*-2-[(2,2,2-trichloro-1-hydroxyethyl)amino]pyrimidine (**22**)

Over a suspension of 2-aminopyrimidine (**8**) (1.000 g, 10.526 mmol) in THF (6.0 mL), 2,2,2-trichloroethane-1,1-diol (4.314 g, 26.318 mmol) was added at room temperature. The resulting suspension was refluxed in THF for 24 hours. After this time, celite was added to the reaction mixture and the solvent was removed under reduced pressure. The residue was purified by column chromatography (Hex:EtOAc, 1:1), yielding *rac*-2-[(2,2,2-trichloro-1-hydroxyethyl) amino]pyrimidine (**22**) (1.367 g, 54% yield) as a white solid.

***R_f_*** = 0.28 (TLC, Hex/EtOAc, 1:1). **^1^H-NMR** (*500 MHz*, *CD_3_OD*) δ: 8.39 (2H, d, *J* = 4.9 Hz), 6.80 (1H, t, *J* = 4.9 Hz), 6.26 (1H, s) ppm. **^13^C-NMR** (*125 MHz*, *CD_3_OD*) δ: 162.4, 159.4, 113.8, 103.7, 84.1 ppm. **LRMS** (*API-ES^+^*): *m/z* 224 (M−18)^+^, 242 (M+H)^+^. **IR** (*Solid*): ν 3298, 3081, 3019, 2970, 2830, 2713, 1589, 1577, 1513, 1459, 1411, 1320, 1253, 1218, 1130, 1071, 998, 981, 910, 802, 788, 685, 647, 634, 598, 549, 511, 464 cm^−1^.

- 1. Synthesis of *rac*-2-[(1,2,2,2-tetrachloroethyl)amino]pyrimidine (**23**)

Over a solution of *rac*-2-[(2,2,2-trichloro-1-hydroxyethyl)amino]pyrimidine (**22**) (414 mg, 1.707 mmol) in dioxane (17.0 mL), SOCl_2_ (0.19 mL, 2.561 mmol) was added at room temperature. The resulting solution was refluxed in dioxane for 30 minutes. After this time, the solvent was removed under reduced pressure, yielding *rac*-2-[(1,2,2,2-tetrachloroethyl)amino]pyrimidine (**23**) (445 mg, 1.707 mmol) as a white solid.

***R_f_*** = 0.15 (TLC, EtOAc/MeOH, 50:1). **^1^H-NMR** *(500 MHz*, *(CD_3_)_2_CO*) δ: 8.55 (2H, d, *J* = 4.8 Hz), 7.85 (1H, d, *J* = 10.9 Hz), 7.19 (1H, d, *J* = 10.9 Hz), 7.01 (1H, t, *J* = 4.8 Hz) ppm. **^13^C-NMR** (*125* *MHz*, *(CD_3_)_2_CO*) δ: 160.7, 159.6, 115.5, 100.7, 79.9 ppm. **LRMS** (*API-ES^+^*): *m/z* 224 (M−36)^+^, 242 (M−36+18)^+^.

- 1. Synthesis of *rac*-2-[(1-{[(1*H*-benzimidazol-2-yl)methyl]amino}-2,2,2-trichloroethyl)amino] pyrimidine (**4**).

Over a solution of *rac*-2-[(1,2,2,2-tetrachloro-1-hydroxyethyl)amino]pyrimidine (**23**) (445 mg, 1.707 mmol) in CH_3_CN (15.0 mL), a suspension of 2-(aminomethyl)benzimidazole dihydrochloride (383 mg, 1.707 mmol) and Et_3_N (1.10 mL, 7.681 mmol) in CH_3_CN (10.0 mL), previously stirred for 15 minutes, was added at room temperature. The reaction mixture was stirred at room temperature for 24 hours. After this time, celite was added to the reaction mixture and the solvent was removed under reduced pressure. The residue was purified by column chromatography (CH_2_Cl_2_:MeOH, 25:1), yielding *rac*-2-[(1-{[(1*H*-benzimidazol-2-yl)methyl] amino}-2,2,2-trichloroethyl)amino]pyrimidine (**4**) (539 mg, 85% yield, 2 steps) as a white solid.

***R_f_*** = 0.32 (TLC, CH_2_Cl_2_/MeOH, 10:1). **^1^H-NMR** (*500* *MHz*, *CD_3_OD*) δ: 8.22 (2H, d, *J* = 4.8 Hz), 7.50-7.46 (2H, m), 7.21-7.18 (2H, m), 6.65 (1H, t, *J* = 4.8 Hz), 5.77 (1H, s), 4.25 (1H, d, *J* = 15.3 Hz), 4.20 (1H, d, *J* = 15.3 Hz). **^13^C-NMR** (*125* *MHz*, *CD_3_OD*) δ: 163.1, 159.1, 154.6, 123.5, 115.7, 113.4, 103.9, 77.6, 45.2 ppm. **LRMS** (*API-ES^+^*): *m/z* 276 (M−95)^+^, 371 (M+H)^+^. **IR** (*Solid*): ν 3422, 3205, 3032, 2921, 2785, 1587, 1578, 1523, 1490, 1446, 1416, 1393, 1357, 1305, 1267, 1212, 1176, 1142, 1117, 1111, 1073, 1036, 993, 846, 838, 809, 797, 785, 737, 718, 664, 642, 626, 593, 529, 491, 479 cm^−1^.

2. **Spectroscopic and spectrometric data**

***O*-(4-Nitrophenyl)-*O*ʹ-[2-(1*H*-2-methyl-5-nitroimidazol-1-yl)ethyl]carbonate (10)**

**[2-(1*H*-2-Methyl-5-nitroimidazol-1-yl)ethyl]carbamate (12)**

***rac*-[2-(1*H*-2-Methyl-5-nitroimidazol-1-yl)ethyl]-*N*-(2,2,2-trichloro-1-hydroxyethyl) carbamate (15)**

***rac*-[2-(1*H*-2-Methyl-5-nitroimidazol-1-yl)ethyl]*-N*-[2,2,2-tricloro-1-(pirimidin-2-ylamino) ethyl]carbamate (1)**

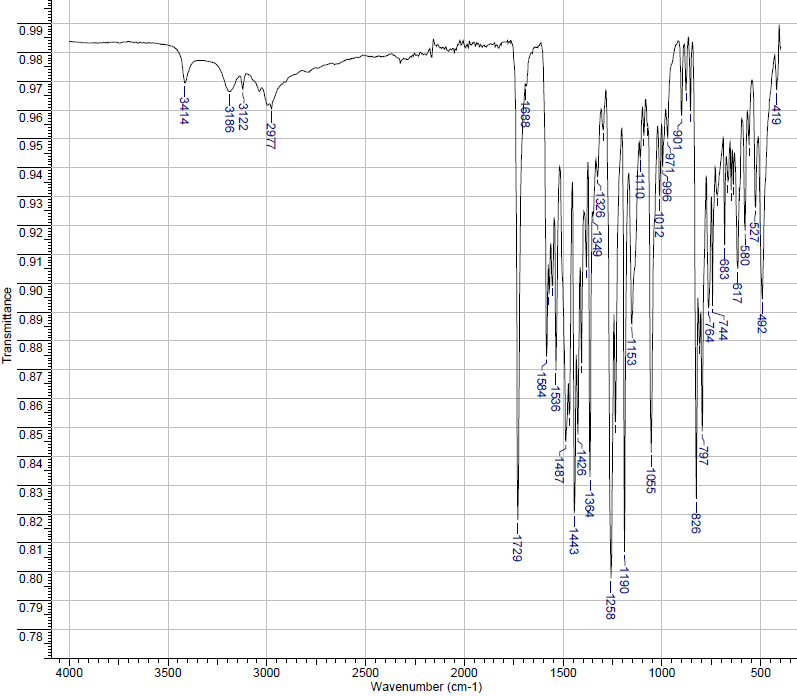


**(Benzyl)carbamate (14)**

**Benzyl-(2,2,2-trichloro-1-hydroxyethyl)carbamate (17)**

***rac*-Benzyl-*N*-[2,2,2-trichloro-1-(pyrimidin-2-ylamino)ethyl]carbamate (2)**

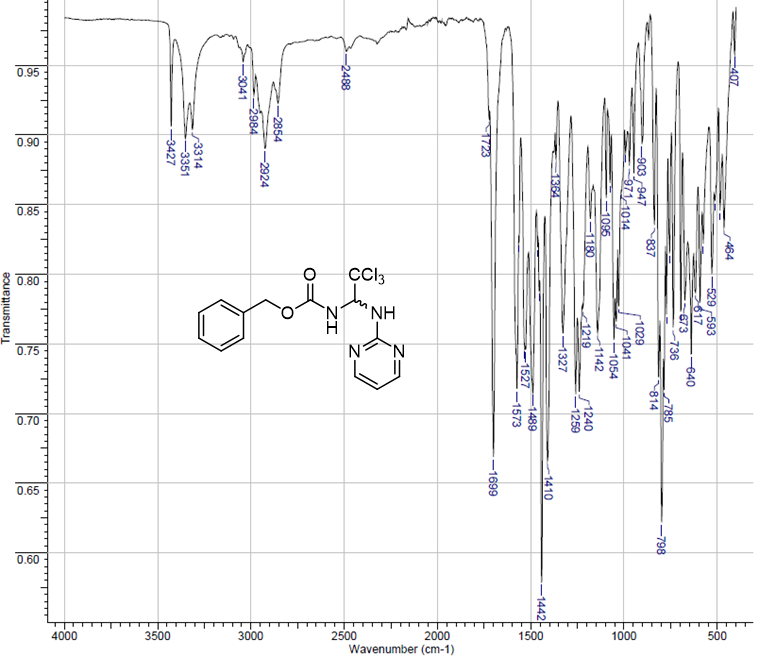


**3-[(*tert*-Butoxycarbonyl)methylamino]propan-1-ol (7)**

***O*-{3-[(*tert*-Butoxycarbonyl)methylamino]propan-1-yl}-*O*'-(4-nitrophenyl)carbonate (11)**

**{3-[(*tert*-Butoxycarbonyl) methylamino]propan-1-yl}carbamate (13)**

***rac*-{3-[(*tert*-Butoxycarbonyl)methylamino]propan-1-yl}-*N*-[2,2,2-trichloro-1-hydroxyethyl] carbamate (16)**

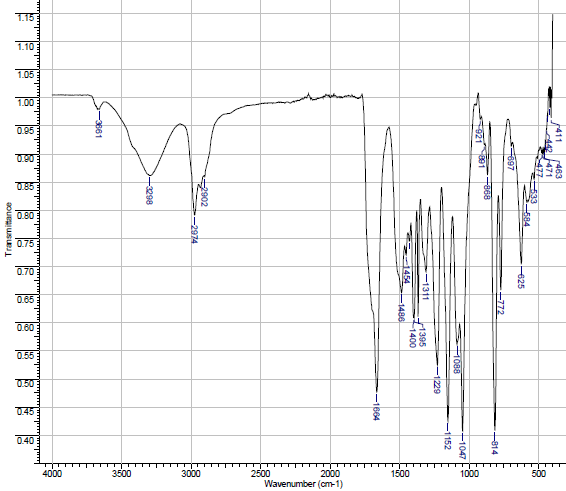

***rac*-{3-[(*tert*-Butoxycarbonyl)methylamino]propan-1-yl}-*N*-[-2,2,2-trichloro-1-(pyrimidin-2-ylamino)ethyl]carbamate (21)**

### ***rac*-[3-(Methylamino)propan-1-yl]-*N*-[2,2,2-trichloro-1-(pyrimidin-2-ylamino)ethyl] carbamate (3)**

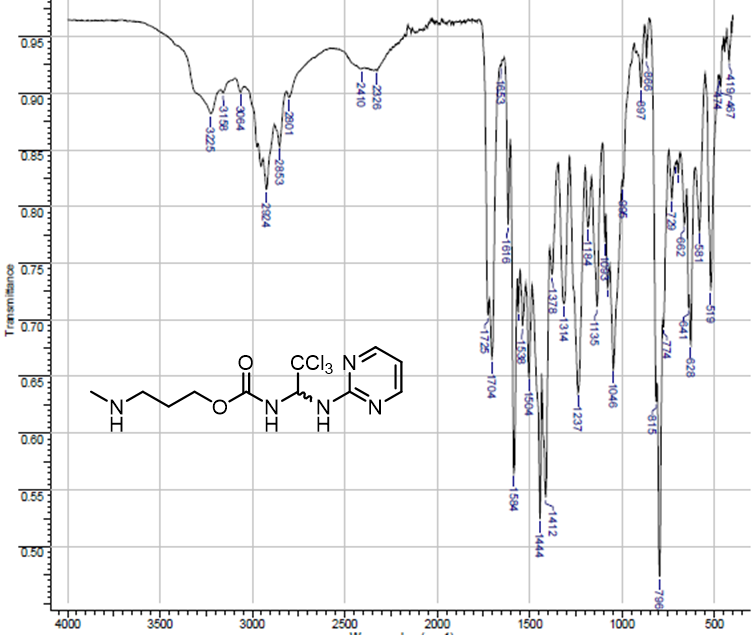


***rac*-2-[(-2,2,2-Trichloro-1-hydroxyethyl)amino]pyrimidine (22)**

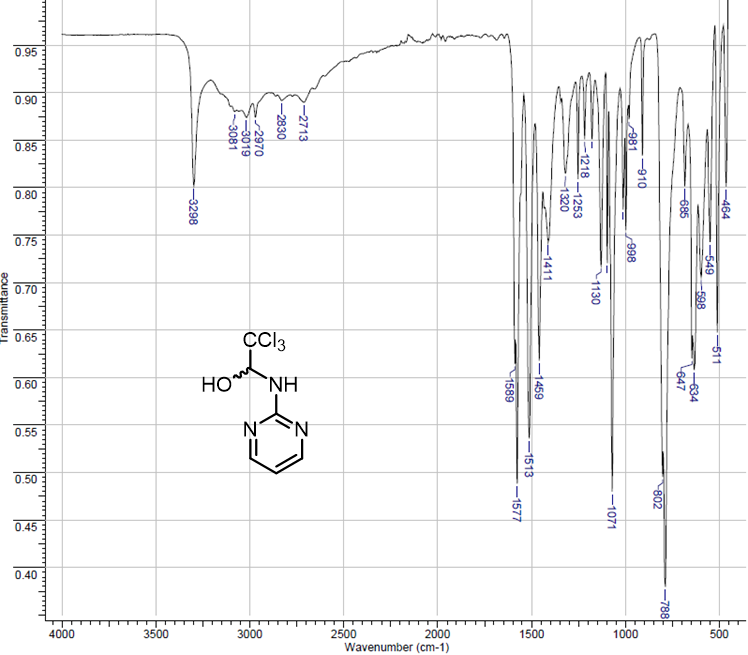


***rac*-2-[(-2,2,2-Trichloro-1-hydroxyethyl)amino]pyrimidine (23)**

***rac*-2-[(1-{[(1*H*-Benzimidazol-2-yl)methyl]amino}-2,2,2-trichloroethyl)amino]pyrimidine (4)**

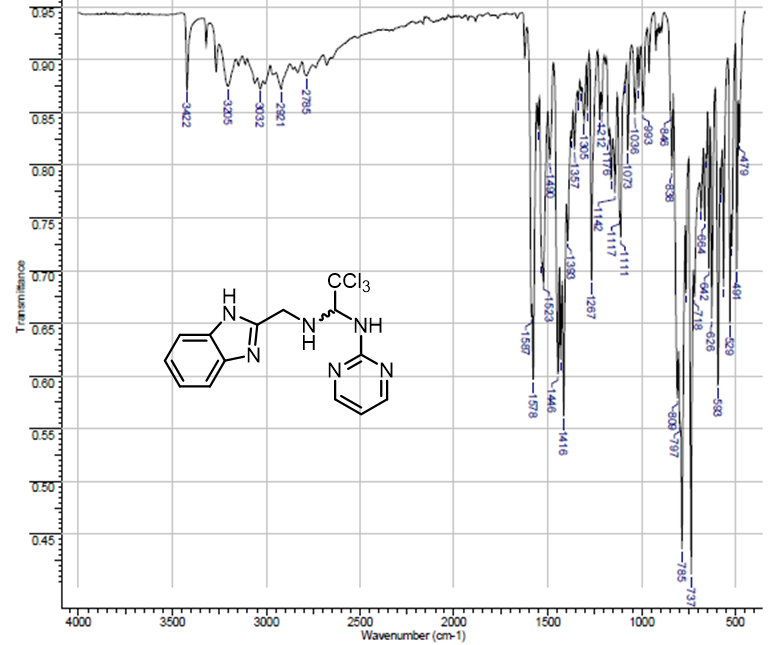


**References**

[13] Sackton, K.L.; Dimova, N.; Zeng, X.; Tian, W.; Zhang, M.; Sackton, T.B.; Meaders, J.; Pfatt, K.L.; Sigoillot, F.; Yu, H.; Luo, X.; King, R.W. Synergistic Blockade of Mitotic Exit by Two Chemical Inhibitors of the APC/C. *Nature*, **2014**, *514*, 646-649. https://doi.org/[10.1038/nature13660](https://doi.org/10.1038/nature13660).

[17] Huang, P.; Le, X.; Huang, F.; Yang, J.; Yang, H.; Ma, J.; Hu, G.; Li, Q.; Chen. Z. Discovery of a Dual Tubulin Polymerization and Cell Division Cycle 20 Homologue Inhibitor Via Structural Modification on Apcin*. J. Med. Chem.* **2020**, *63*, 4685-4700. https://doi.org/10.1021/acs.jmedchem.9b02097.

[38] For the preparation of compound **7** from 3-aminopropan-1-ol, see: Flack, T.; Romain, C.; White, A.J.P.; Haycock, P.R.; Barnard, A. Design, Synthesis, and Conformational Analysis of Oligobenzanilides as Multifacial α-Helix Mimetics. *Org. Lett.* **2019**, *21*, 4433−4438. https://doi.org/10.1021/acs.orglett.9b01115.

[39] Inaloo, I.D.; Majnooni, S.; Esmaeilpour, M. Superparamagnetic Fe_3_O_4_ Nanoparticles in a Deep Eutectic Solvent: An Efficient and Recyclable Catalytic System for the Synthesis of Primary Carbamates and Monosubstituted Ureas. *Eur.* *J. Org. Chem.* **2018**, 3481-3488. https://doi.org/10.1002/ejoc.201800581.

[40] Meyer, H.; Beck, A.K.; Sebesta, R.; Seebach, D. Benzyl Isopropoxymethyl Carbamate – An Aminomethylating Reagent for Mannich Reactions of Titanium Enolates. *Organic Syntheses* **2008**, *85*, 287-294. https://doi.org/10.1002/0471264229.os085.29.

[41] Svestka, D.; Otevrel, J.; Bobal, P. Asymmetric Organocatalyzed Friedel–Crafts Reaction of Trihaloacetaldehydes and Phenols Adv. *Synth. Catal.* **2022**, *364*, 2174-2183. https://doi.org/10.1002/adsc.202200180.

[42] Yan, Y-H.; Li, W.; Chen, W.; Li, C.; Zhu, K-R.; Deng, J.; Dai, Q-Q.; Yang, L-L.; Wang, Z.; Li, G-B. Structure-Guided Optimization of 1*H*-Imidazole-2-Carboxylic Acid Derivatives Affording Potent VIM-Type Metallo-β-Lactamase Inhibitors. *Eur. J. Med. Chem.* **2022**, *228*, 113965. https://doi.org/10.1016/j.ejmech.2021.113965.

[43] Pestellinii, V.; Vitil, G.; Nannicinii, R.; Borsinil, F.; Furio, M.; Leccii, A.; Volterra, G.; Meli, A. 11-Alkoxy-Dibenzo[b,e]azepin-6-ones with Anti-Convulsant Activity. *Eur. J. Med. Chem.* **1988**, *23*, 473-476.

[44] Simonetti, G.; Boga, C.; Durante, J. Micheletti, G.; Telese, D.; Caruana, P.; Luserna di Rorà, A.G.; Mantellini, F.; Bruno, S.; Martinelli, G.; Calonghi, N. Synthesis of Novel Tryptamine Derivatives and Their Biological Activity as Antitumor Agents. *Molecules* **2021**, *26*, 683-696. https://doi.org/10.3390/molecules26030683.

[45] Anet, P.F.; Yavari, I. Nitrogen Inversion in Piperidine. *J. Am. Chem. Soc.*, **1977**, *99*, 2794-2796. <https://doi.org/10.1021/ja00450a064>.

[46] Kuprianowicz, M.; Kaźmierczak, M.; Wójtowicz-Rajchel, H. The Nitrogen Inversion in Fused Isoxazolidinyl Derivatives of Substituted Uracil: Synthesis, NMR and Computational Analysis. *Struct. Chem*. **2016**, *27*, 1265-1278. https://doi.org/10.1007/s11224-016-0755-4.
