# Supplementary material for: Targeting APC/C Ubiquitin E3-Ligase Activation with Pyrimidinethylcarbamate Apcin Analogues for the Treatment of Breast Cancer
Source: Biomolecules. 2024 Nov 12;14(11):1439. doi: 10.3390/biom14111439 (PMC11591962; doi:10.3390/biom14111439)
Supplement: Supplementary file 1 [file biomolecules-14-01439-s001.zip › biomolecules-3253073-Supplementary Table S1.docx]

**Supplementary Table 1**. Per-residue Van der Waals (VdW) and electrostatic contributions (expressed in kcal/mol) of APN and the new APN analogues.

| **Molecule ID** | | **APN** | **Me-APN** | **Bzn-APN** | **Cbz-APN** |
| --- | --- | --- | --- | --- | --- |
| **Glide Score** | | -6.12 | -7.67 | -6.57 | -6.33 |
| Val216 | VdW | -4.10 | -3.27 | -3.66 | -4.19 |
|  | Electrostatic | -0.13 | 0.21 | 0.76 | -0.43 |
| Trp209 | VdW | -4.10 | -3.27 | -3.66 | -3.61 |
|  | Electrostatic | 0.36 | 0.55 | -0.33 | 0.53 |
| Tyr207 | VdW | -6.54 | -4.72 | -3.92 | -5.77 |
|  | Electrostatic | -0.64 | -4.45 | 0.23 | -0.43 |
| Leu202 | VdW | -2.94 | -2.66 | -2.06 | -2.28 |
|  | Electrostatic | 0.16 | -0.24 | 0.34 | 0.06 |
| Val200 | VdW | -2.44 | -2.10 | -1.17 | -2.37 |
|  | Electrostatic | -0.17 | -0.58 | 0.27 | -0.01 |
| Glu180 | VdW | -0.11 | -2.57 | -1.68 | -0.10 |
|  | Electrostatic | -1.05 | -28.61 | -13.05 | -0.62 |
| Pro179 | VdW | -3.33 | -2.57 | -1.68 | -1.69 |
|  | Electrostatic | -0.92 | -1.72 | -0.24 | 0.05 |
| Ala178 | VdW | -1.06 | -1.37 | -1.42 | -1.31 |
|  | Electrostatic | 0.37 | -1.20 | -1.41 | -0.03 |
| Asp177 | VdW | -3.46 | -3.16 | 0.62 | -3.59 |
|  | Electrostatic | -4.47 | -23.72 | -42.09 | -6.95 |
| Leu176 | VdW | -5.42 | -5.67 | -3.96 | -5.44 |
|  | Electrostatic | -0.87 | -0.26 | 2.18 | -0.67 |
