# Supplementary figures and images for: Targeting APC/C Ubiquitin E3-Ligase Activation with Pyrimidinethylcarbamate Apcin Analogues for the Treatment of Breast Cancer
Source: Biomolecules. 2024 Nov 12;14(11):1439. doi: 10.3390/biom14111439 (PMC11591962; doi:10.3390/biom14111439)

Original WB blot images time-course experiment. Time expressed in minutes.

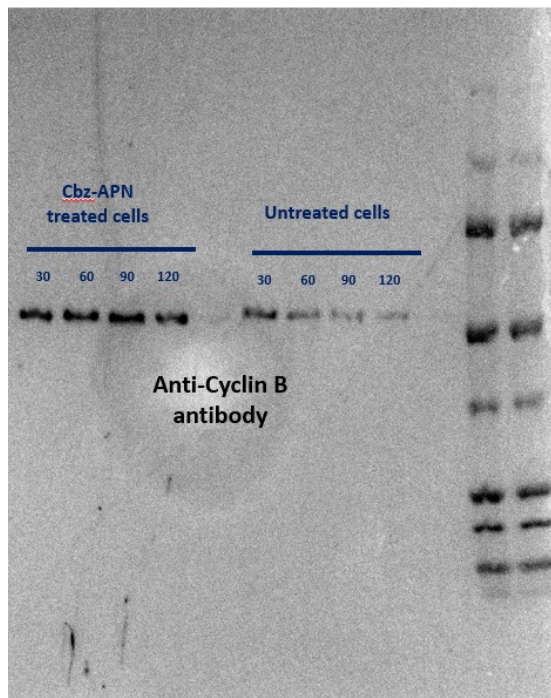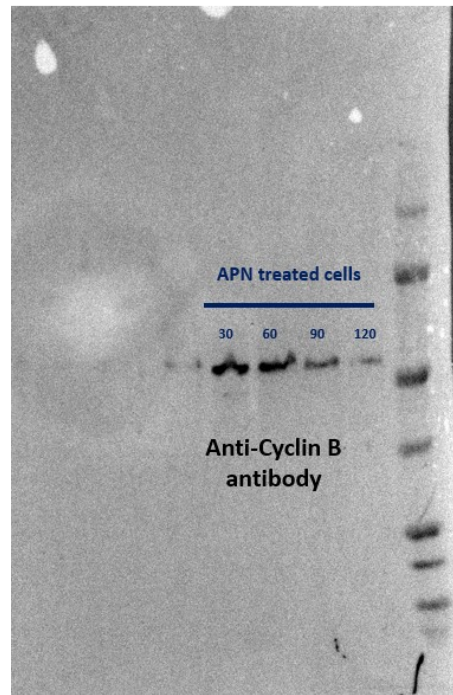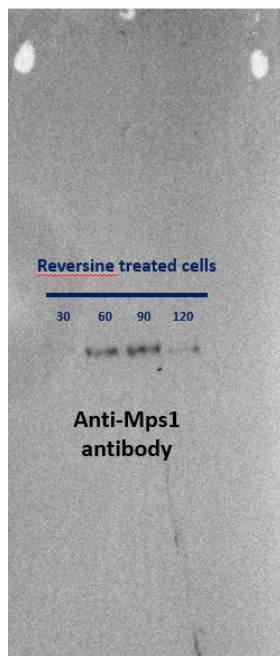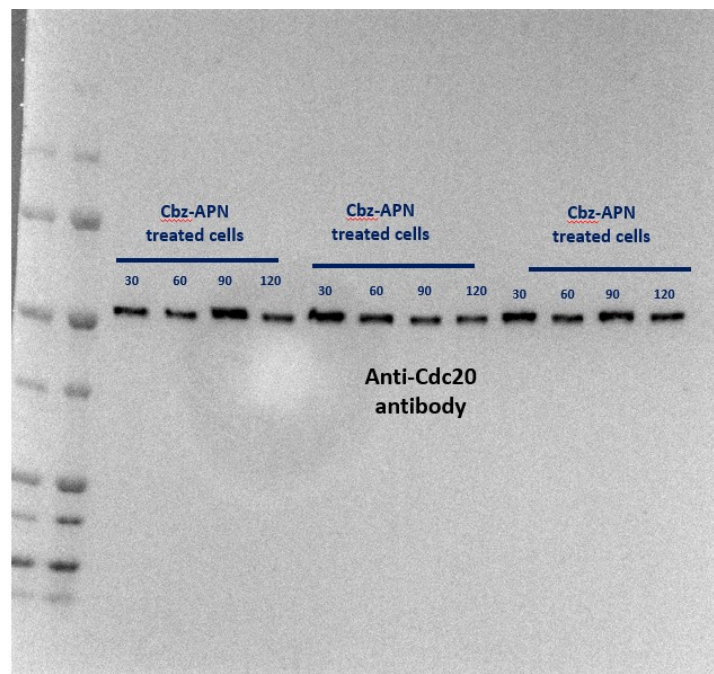

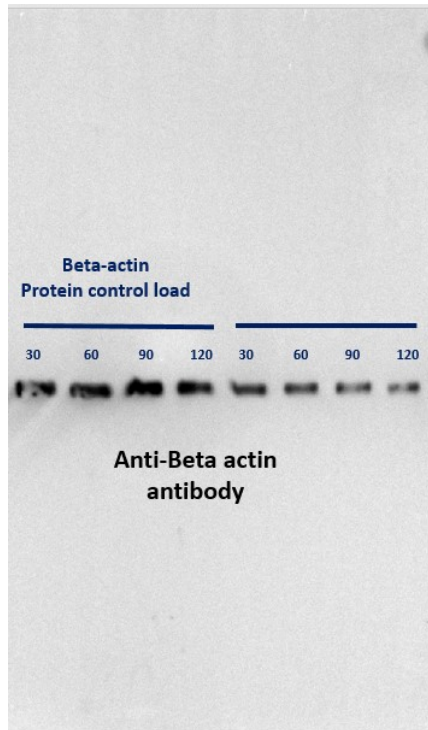

Supplement: Supplementary file 1 [file biomolecules-14-01439-s001.zip › biomolecules-3253073-Supplementary File S1.pdf]
